# Corrosion Resistance and Apatite-Forming Ability of Composite Coatings formed on Mg–Al–Zn–Ca Alloys

**DOI:** 10.3390/ma12142262

**Published:** 2019-07-14

**Authors:** Anawati Anawati, Hidetaka Asoh, Sachiko Ono

**Affiliations:** 1 Department of Physics, Faculty of Mathematics and Natural Sciences, Universitas Indonesia, Depok 16424, Indonesia; 2 Department of Applied Chemistry, Kogakuin University, 2665-1 Nakano, Hachioji, Tokyo 192-0015, Japan

**Keywords:** magnesium, microstructure, coating, corrosion, polarization, apatite

## Abstract

The properties of composite coatings formed by plasma electrolytic oxidation (PEO) were affected by the alloy composition. The corrosion resistance and apatite-forming ability of PEO coatings formed on Mg–6Al–1Zn–xCa alloys with a variation of Ca content were investigated. Potentiodynamic polarization and electrochemical impedance spectroscopy (EIS) measurements showed an order magnitude improvement of corrosion resistance in the AZ61 alloy as a result of the coating. A higher enhancement in polarization resistance was obtained in the Mg–6Al–1Zn–1Ca and Mg–6Al–1Zn–2Ca alloys due to thicker coatings were formed as a result of the incorporation of calcium oxide/hydroxide. However, the underlying substrates were more prone to localized corrosion with increasing Ca content. The microstructure investigation revealed an enlargement in precipitates (Al_2_Ca, Mg_2_Ca) sizes with increasing Ca content in the alloys. The growth of larger size precipitates increased the danger to micro galvanic corrosion. Apatite layers were formed on all of the coatings indicating high apatite-forming ability, but the layers formed on the Mg–6Al–1Zn–1Ca and Mg–6Al–1Zn–2Ca alloys contained higher Mg, possibly due to the accumulation of corrosion product, than that on the Mg–6Al–1Zn alloy. The alloying element Ca should be limited to 1 wt.% as the excess tended to degrade the corrosion resistance and apatite-forming ability of the PEO coating.

## 1. Introduction

The use of magnesium (Mg) and its alloys for biodegradable materials rely on the surface treatment such as plating, coating, and anodizing, due to the high corrosion rate of Mg in aqueous environments [1,2,3]. Among the available techniques, plasma electrolytic oxidation (PEO) becomes famous for corrosion protection of Mg alloys considering its flexibility to coat complex geometry and environmentally friendly process [4,5]. The PEO coating that forms as a result of high-voltage (hundreds volt) anodization in an alkaline electrolyte, with the incorporation of the electrolyte species, provides superior corrosion and wear resistance [6,7,8]. A ceramic-like composite layer consisting of crystalline and amorphous oxides is developed under the exposure of a high-temperature plasma discharge. The corrosion resistance of the PEO coating depends on the processing parameters [9], alloy composition [10], and the type of electrolyte used [11]. Among various alkaline electrolytes, the PEO coating formed in phosphate-based solution exhibited the highest corrosion resistance due to the significant formation of crystalline magnesium phosphate [12]. Magnesium phosphate is a biocompatible compound and has attracted much attention during the past few years as a material for bone replacement [13]. However, both the in vitro [7,14] and in vivo [15,16] tests have indicated that the deposition of bone mineral apatite (Ca_10_(PO_4_)_6_) on the PEO coating was limited. The apatite-forming ability defines the bioactivity of biomaterials.

Various methods have been proposed to accelerate the bioactivity of the PEO coating in physiological solution such as by coating with apatite [17,18], incorporating Ca [19,20] or apatite [21,22] in the coating, and introducing Ca as an alloying element [23]. The unstable Ca compounds easily dissolves in the electrolyte during plasma discharge and therefore adding Ca in the electrolyte bath is not beneficial. Incorporation of apatite in the PEO coating improved the corrosion resistance, but long-term immersion in simulated body fluid (SBF) indicated no further growth of the apatite [19,20]. Apart from the coating modification during the PEO process, the effect of Ca as an alloying element in Mg in modifying the bioactivity of the coating has not been thoroughly investigated. Our previous result [23] showed an increase in the bioactivity of the PEO coating on Mg–6Al–0.26 Mn–xCa alloys with increasing Ca concentration in the alloys. An embodiment of Ca in the coating and its presence in the alloy accelerated the growth of apatite in simulated body fluid (SBF). However, short-term (8 min) grown coatings on Mg–6Al–1Zn–xCa alloys revealed no bioactivity in SBF and significantly high corrosion rate [24]. The reason for such behavior was still unclear. The alloying element Ca might have different effect on the microstructure, and hence the resulting PEO film of the Mg–Al–Zn alloys as compared to that of the Mg–Al–Mn alloys. The development of an Mg based-biodegradable implant included Zn as alloying element to improve its mechanical strength [25,26,27]. This work aims to throw light on explaining the corrosion behavior and apatite-forming ability of the coatings formed on the Mg–6Al–1Zn–xCa alloys as a result of alteration in the microstructure with a variation of Ca content.

## 2. Materials and Methods 

### 2.1. Specimen Preparation

Rolled plates of Mg–6Al–1Zn–xCa alloys with the composition listed in Table 1 were used as substrates. For the sake of simplicity, the alloy was further designated as AZ61, AZX611, and AZX612 for the alloys containing 0, 1, and 2 wt.% Ca, respectively. The plate was cut into 5 cm^2^ working area. For surface observation, the specimen was embedded in a resin and then ground to 1200-grit silicon carbide (SiC) paper. The specimen was then degreased in acetone in an ultrasonic bath for 3 min. The etching treatment was applied on the specimen using 4 vol.% HNO_3_ in ethanol for 20 s to reveal the microstructure.

### 2.2. Plasma Electrolytic Oxidation

To dissolve the natural oxide layer on the surface, the specimen was dipped in a mixed acid solution of 8 vol.% HNO_3_-1 vol.% H_3_PO_4_ for 20 s, and then in 5 wt.% NaOH solution at 80 °C for 1 min. The PEO coating was developed under a galvanostatic mode at 200 Am^−2^ in 0.5 mol·dm^−3^ Na_3_PO_4_ solution at 25 °C for 20 min. A regulated DC power supply was used as the current source. The specimen was placed as an anode while a pair of carbon rod was used as cathode. The voltage output was measured by digital multimeter bench from Keithley series 2700. A coating thickness gauge of dual type (SME-1) from Sanko was used for determining the coating thickness.

### 2.3. Electrochemical Corrosion 

The electrochemical corrosion measurement was performed by conducting potentiodynamic polarization and electrochemical impedance spectroscopy (EIS) tests in a physiological solution 0.9 wt.% NaCl solution at 37 °C based on ASTM G5 [28]. A defined surface with an area of 2.5 cm^2^ was exposed to the solution. The electrochemical tests were performed by using a potentiostat instrument from IviumStat. Pt wire was used as a counter electrode, and silver chloride (Ag/AgCl) was used as a reference electrode. The electrochemical cell condition and arrangement were similar to as reported earlier [24]. Potentiodynamic polarization test was conducted from 100 mV below open circuit potential (OCP) and terminated when the current output reached 30 mA at a sweep rate of 1 mV·s^−1^. The corrosion potential and current density were estimated by using Tafel extrapolation. The EIS was performed on the uncoated substrates at an open circuit potential (OCP) over a frequency ranging from 10^−2^ Hz to 10^4^ Hz. Prior to the tests, the specimen was left at an OCP for 40 min to stabilize the potential. The coated specimen did not give spectra at low frequency and therefore was analyzed at frequency range 10^2^ to 10^7^ Hz.

### 2.4. Apatite-Forming Ability Test

The apatite-forming ability test was performed by immersing the specimens individually in SBF at 37 °C with a surface-to-volume ratio of 20 mL/cm^2^ for 14 days. An SBF10, with the ionic concentration shown in Table 2, was used. The solution preparation followed the Reference [29]. The fresh solution pH was adjusted to 7.4 at 37 °C. The solution was replaced after 1, 3, 5, 7, 10, and 12 days, and was collected each time after each replacement for further analysis. The Mg concentration dissolved in the solution was analyzed by using the titration method. A digital titration kit from Hach, which included a digital titrator, buffer solutions, and indicators, was used. The solution was titrated with 0.08 M of sodium ethylenediaminetetraacetic acid (EDTA) using a digital titrator. The method was able to detect Mg concentrations within a 1 ppm margin of error.

### 2.5. Surface Analyses

The surface microstructure and the elemental composition was studied by an energy dispersive X-ray spectroscopy (EDS, JEOL EX-54175JMU, Tokyo, Japan) attached to the SEM (JEOL JSM-6380LA, Tokyo, Japan). The crystalline phases in the specimens were analyzed using thin coating X-ray diffraction analysis (TF-XRD, Rint 2000 Rigaku type, Tokyo, Japan) at an incident angle of 1°. The phases existed in the specimens were analyzed by indexing the peaks in the XRD pattern by referring to the JCPDS cards. Depth profile analysis on the coatings was performed on a circular area with a diameter of 4 mm using glow-discharge optical emission spectroscopy (GDOES, Jobin-Yvon JY5000RF, Horiba, Ltd., Kyoto, Japan). Sputtering was done using Ar^+^ ion at 40 W. 

## 3. Results

### 3.1. Alloy Microstructure

**Figure 1:**
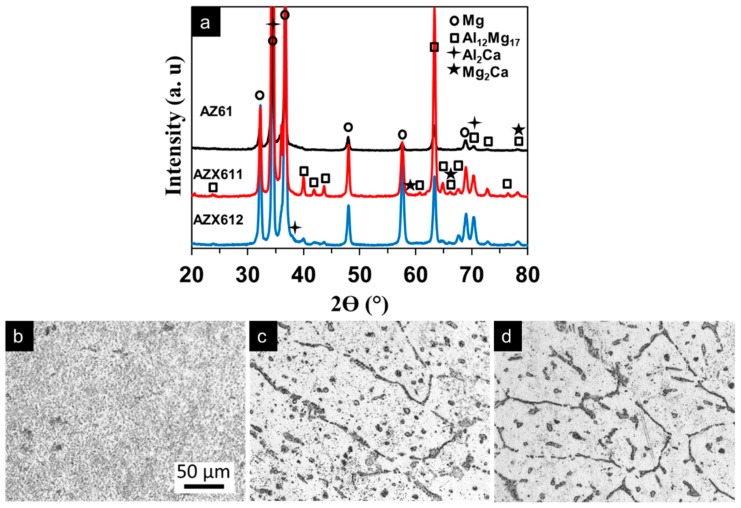
(**a**) X-ray diffraction pattern and surface microstructure of (**b**) AZ61, (**c**) AZX611, and (**d**) AZX612 alloys. The scale bar in image (b) applied to images (c) and (d).

Figure 1 shows the X-ray diffraction (XRD) pattern of AZ61, AZX611, and AZX612 alloys and the optical microscope images showing the microstructure of the alloys. All of the alloys consisted of primary α-Mg phase and secondary β-phase (Al_12_Mg_17_). Precipitation of Ca-containing intermetallic, Al_2_Ca and Mg_2_Ca, was detected in the AZX611 and AZX612 alloys. The peaks for Al_2_Ca and Mg_2_Ca phases were quite small and often overlapped with the peaks for other phases. As opposed to the microstructure of AM60 alloy which contained a low number of precipitates [23], the Mg matrix of the AZ61 alloy exhibited an ultrafine equiaxed grain with the average grain size ~3 µm × 5 µm decorated by numerous spherical intermetallic Al_12_Mg_17_ phase with diameters approximately 0.5 µm as shown in Figure 1b. The presence of 1 wt.% Ca expanded the grain size about an order of magnitude, but no further enlargement with increasing Ca content to 2 wt.%. The XRD pattern of AZX611 and AZX612 alloys displayed a higher intensity in the Mg peaks confirming larger metallic grain sizes than that of the base alloy. The equiaxed grains size in both AZX611 and AZX612 alloy was approximately 50 µm × 250 µm. The grain boundaries became thicker with increasing Ca content in the alloys due to segregation of the intermetallic precipitates (Figure 1c,d). The XRD pattern of Ca-containing alloys revealed some additional β-phase peaks emerged at 23.7°, 40.0°, 42.0°, 43.7°, 61.6°, 63.3°, 64.9°, 66.4°, and 67.8° indicating a higher amount of β phase existed in the alloys.

**Figure 2:**
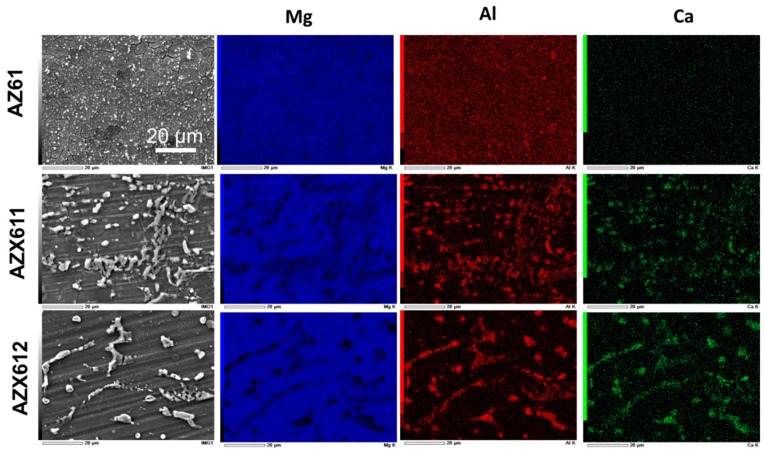
Plane-view SEM images and the corresponding EDS maps for Mg (blue), Al (red), and Ca (green), of AZ61, AZX611, and AZX612 alloys. The scale bar applied to all images.

Figure 2 shows the SEM images and the corresponding EDS maps for the elements Mg, Al, and Ca, showing the element composition of the three alloys. In the maps, blue represents Mg, red represents Al, and the green represents Ca. The AZ61 alloy shows a high density of precipitates on its surface. The maps displayed a nearly uniform blue and red maps corresponded to the β precipitate smeared in the matrix. The size of precipitates became larger with increasing Ca content in the alloys, and the matrix became clearer from precipitates. The maps for AZX611 and AZX612 showed that the intermetallic consisted of Mg, Al, and Ca. This analysis proved the unambiguous presence of Al_2_Ca and Mg_2_Ca precipitates besides the β phase in both AZX611 and AZX612 alloys. The amount of Al_2_Ca phase dominated over Mg_2_Ca phase. 

### 3.2. PEO-Coating Structure and Composition

**Figure 3:**
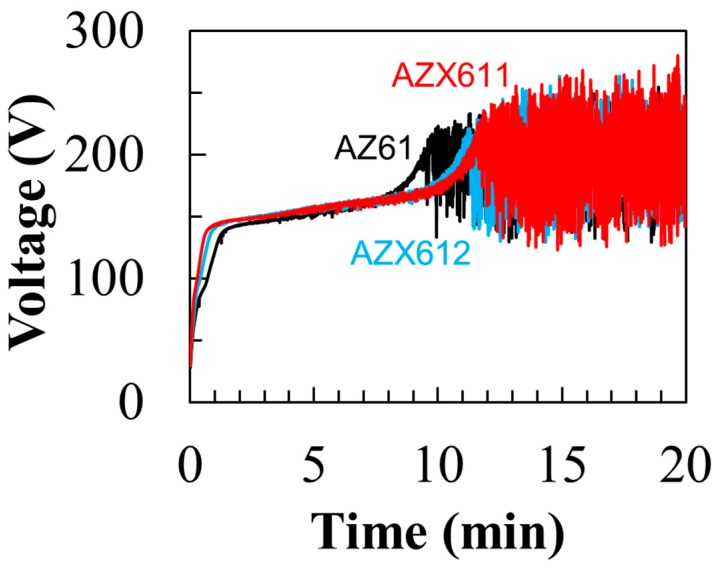
Voltage–time curves during 20 min anodization of AZ61, AZX611, and AZX612 alloys in 0.5 M Na_3_PO_4_ solution at 25 °C.

The voltage–time curves recorded during the PEO coating on the three alloys are depicted in Figure 3. Above the breakdown voltage (90 V), plasma discharge was developed at a few spots on the specimen surface, indicating the beginning of the electrolytic process. After the discrete plasma formed uniformly on the surface, the voltage stabilized at ~150 V. Strong plasma discharge began to occur at a critical voltage after about 9 min where large oscillation in the voltage (150–250 V) was attained. The presence of 1 wt.% Ca in the alloys shifted the critical voltage towards a longer time of approximately 2 min; however, the curve did not shift any further when the amount of Ca was increased to 2 wt.%. 

**Figure 4:**
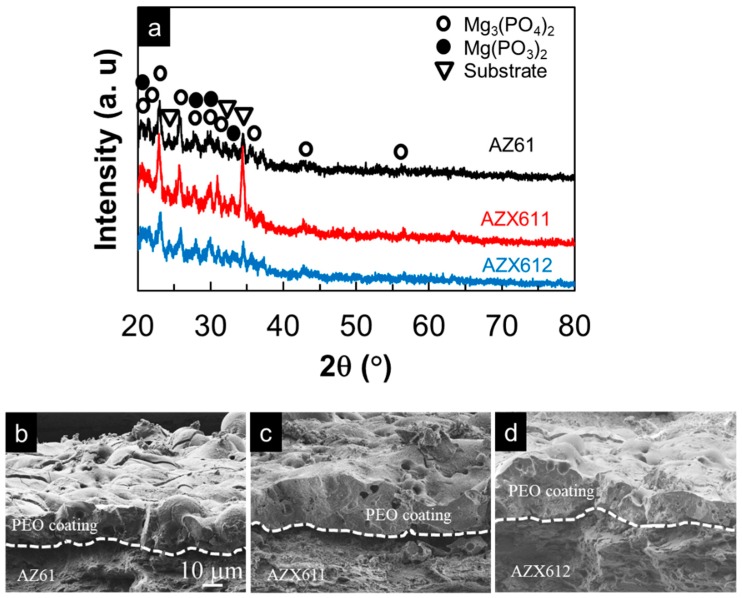
(**a**) X-ray diffraction pattern of plasma electrolytic oxidation (PEO) coatings and the corresponding cross-section FE-SEM images of the coating on (**b**) AZ61, (**c**) AZX611, and (**d**) AZX612 resulting from 20 min anodization in 0.5 M Na_3_PO_4_ solution at 25 °C. The scale bar applied to all images.

Figure 4 displays the XRD pattern of the resulting coatings formed on the three alloys and the cross-sectional FE-SEM images of the coatings. Most of the peaks for substrates no longer appeared in the XRD pattern of the coated specimens indicating an excellent blocking effect of the coatings from the X-ray. The composition of the coatings formed on the three alloys were similar, which were composed of both crystalline and amorphous phases as shown by the appearance of the broad peak between 20° and 40°, which attributed to the amorphous phase, and the small peaks inside the broad peak corresponded to the crystalline oxide phases of Mg_3_(PO_4_)_2_ and Mg(PO_3_)_2_. The coatings that formed on all of the specimens exhibited a similar uneven structure, as shown in Figure 4b–d. Pores and cracks, which are the footprint of PEO coating, were observed on both the surface and inner part of the coating. Fine cracks were developed during the PEO process, while heavy cracks occurred unintentionally during specimen preparation. The coating formed on Ca-containing alloys was slightly thicker than that of the base alloy. In agreement with the cross-section images, the average coating thickness measured by coating thickness gauge were 25, 32, and 30 µm for the coating formed on AZ61, AZX611, and AZX612, respectively. The XRD analysis did not detect the presence of Ca in the coating, similar to the earlier result on AM60 [23]. The Ca compounds may have been present in an amorphous state. 

**Figure 5:**
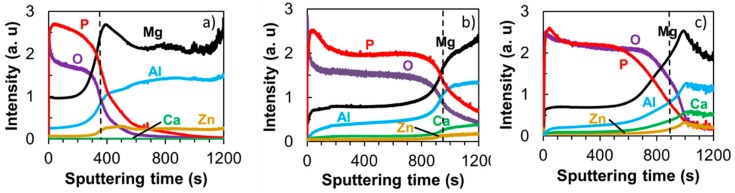
Glow-discharge optical emission spectroscopy (GDOES) elemental depth profiles of PEO coatings on (**a**) AZ61, (**b**) AZX611, and (**c**) AZX612 specimens after anodization in 0.5 mol·dm^−3^ Na_3_PO_4_ solution at 25 °C for 20 min. The dashed lines indicate the oxide–metal interface.

The coatings composition was further analyzed by depth profile GDOES, and the results are displayed in Figure 5. The dashed line marks the coating–metal interface at which the O and Mg profiles crossed each other. All of the coatings were composed of Mg, P, O, and Al. Confirming XRD results, the primary oxide phases in the coatings consisted of Mg–O compounds. The GDOES profile suggested the presence of other oxide phases, including MgO and Al_2_O_3_ in the amorphous state. The incorporation of Ca in the coating of AZX611 and AZX612 alloys was confirmed by the Ca profiles in Figure 5b,c. The intensities of Ca signal in the AZX611 and AZX612 coatings was about half of their bulk values, which verified the presence of calcium oxide/hydroxide phase in the coatings. The coating-metal interface shifted twice towards longer sputtering times from 400 s for AZ61 to 950 s for AZX611 and 900 s for AZX612 specimens. The shift indicated an increase in coating thickness as the thicker coating needs longer sputtering time to reach the bulk substrate. 

### 3.3. Electrochemical Corrosion

**Figure 6:**
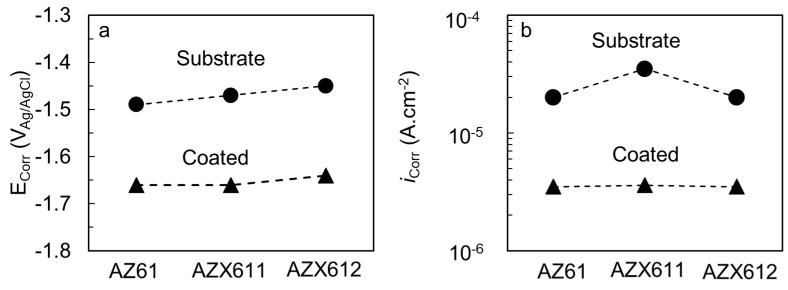
(**a**) Corrosion potentials and (**b**) corrosion current densities of the substrates and coated specimens of AZ61, AZX611, and AZX612 alloys.

The corrosion behavior of the PEO coating was investigated by potentiodynamic polarization test in 0.9 wt.% NaCl solution at 37 °C. Figure 6 shows the corrosion potential and current densities data obtained from the polarization curves of the specimens before and after coating. The corrosion potentials of the substrates became slightly nobler from −1.49 to −1.45 V_Ag/AgCl_ with increasing Ca content in the alloys. The behavior was preserved after coating that the corrosion potential increased with Ca content in the alloys from −1.66 to −1.64 V_Ag/AgCl_ (Figure 6a). The corrosion current densities of the coated specimens were 10 times lower than that of the substrates. The substrates exhibited corrosion current densities in the range of 2.0 × 10^−5^ to 3.5 × 10^−5^ A·cm^−2^ while the coated specimens were in the range of 3.5 × 10^−6^ to 3.6 × 10^−6^ A·cm^−2^. Depression of corrosion potential, which was accompanied by a reduction in corrosion current densities as a result of PEO coating, indicated an improvement in corrosion resistance of the alloys. The inhibition of cathodic reaction on the surface suppressed the corrosion potential of the coated specimens to the negative direction. 

**Figure 7:**
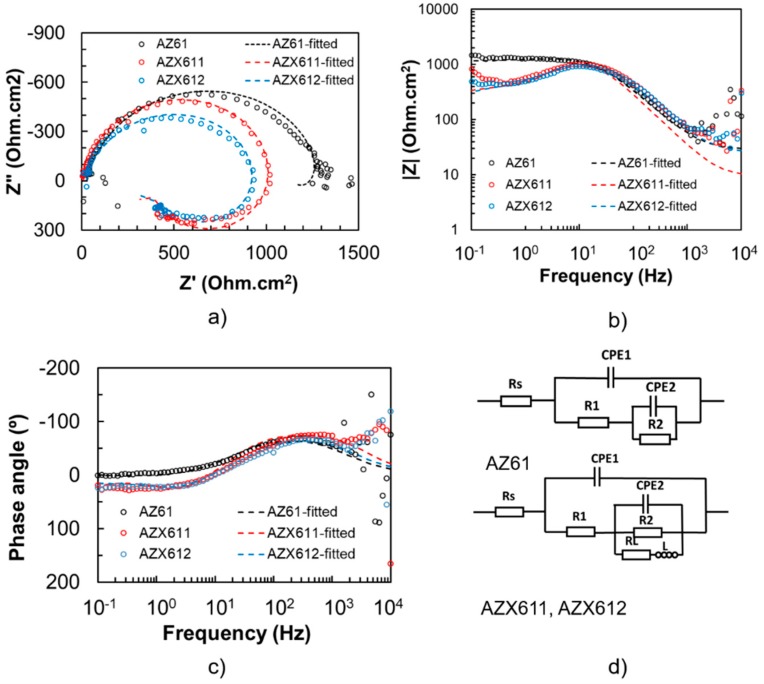
Electrochemical impedance spectra of uncoated specimens of AZ61, AZX611, and AZX612 specimens: (**a**) Nyquist plots, (**b**) bode plots, and (**c**) phase plots, and (**d**) the equivalent circuit.

**Figure 8:**
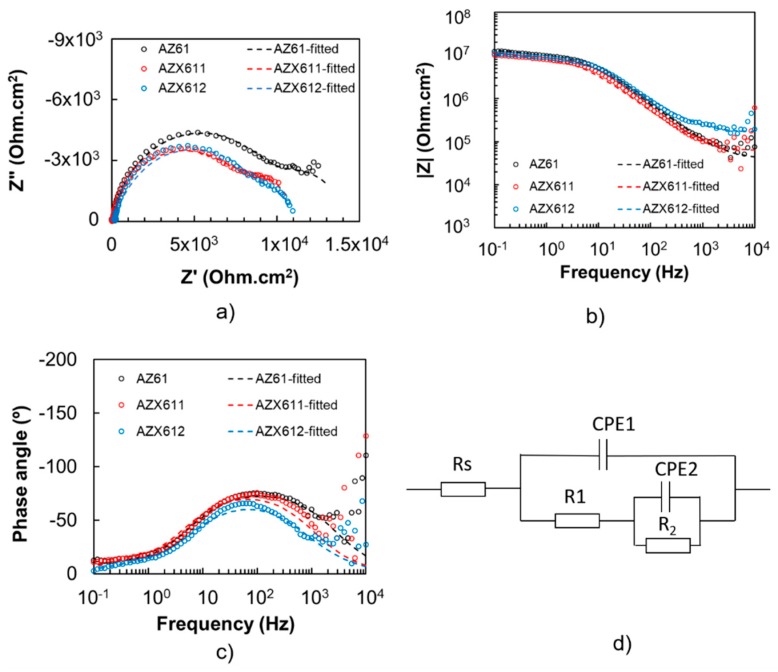
Electrochemical impedance spectra of coated specimens of AZ61, AZX611, and AZX612 specimens: (**a**) Nyquist plots, (**b**) bode plots, and (**c**) phase plots, and (**d**) the equivalent circuit.

The coating resistance was evaluated by EIS measurement in 0.9 wt.% NaCl solution at 37 °C. The results are displayed in Figure 7 for uncoated, and Figure 8 for the coated, specimens. The fitted parameters obtained from the EIS data are listed in Table 3. The n1 and n2 refers to constant phase for CPE1 and CPE2, respectively. The maximum n value is 1. The closer to 1 the n1 and n2 value is, indicates more capacitive behavior. The polarization resistance (R1) of the coated specimens was an order of magnitude higher than that of their substrates. The base alloy exhibited the highest polarization resistance in both uncoated and coated conditions relative to the Ca-containing alloys. The impedance spectrum of AZ61 substrate exhibited one apparent capacitive loop, while the Ca-containing alloys showed an additional inductive loop at low frequency. The corresponding inductive loop is an indication of localized corrosion [5,8]. The presence of inductive loop lowered the n2 values for AZX611 and AZX612 specimens compared to the AZ611 specimen. The presence of a constant phase element (CPE) in parallel with a resistance indicates the presence of a faradaic reaction (charge transfer) and a non-Faradaic reaction (charge accumulation at the interface) occurring at the interface. All of the three PEO coatings exhibited a similar trend of impedance spectrum. The L and R_L_ loops did not exist in the model for the PEO coating (Figure 8), indicating high resistance to metal dissolution. Moreover, a much higher impedance at low and medium frequencies of the Bode plot and an enlarged diameter of the semicircle in the Nyquist plot of the coated specimens are evidence of remarkable higher corrosion resistance than that of the substrates. Moreover, the capacitance values of the coated specimens were a hundred times lower than that of the substrates. The effect of the alloying Ca in the coating impedance was displayed by the Bode plot in Figure 8. The impedance at high frequencies, which represents the outer PEO coating of all the coated specimens, was similar. However, the impedance at low frequencies displayed slightly lower impedance than that of the AZ61 specimen. The results implied that the coating–metal interface of the Ca-containing specimens was more prone to localized corrosion than that of the base specimen.

### 3.4. Apatite-Forming Ability 

**Figure 9:**
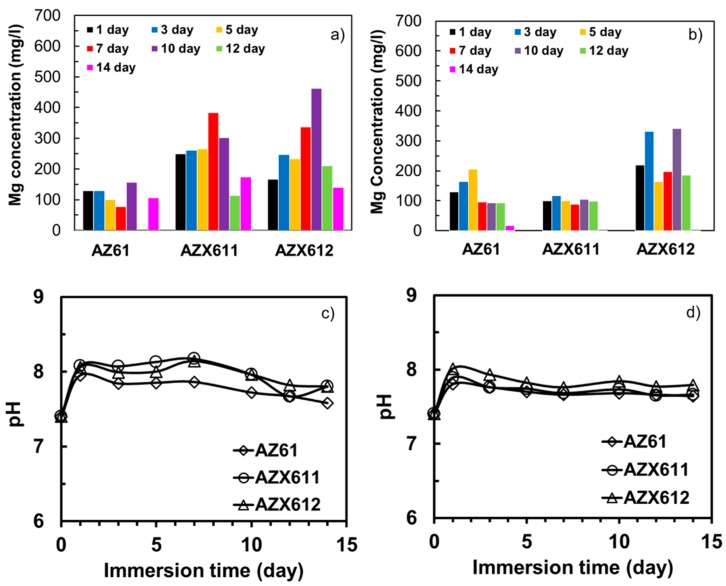
Concentration of Mg dissolved during immersion testing in SBF of the (**a**) substrates, (**b**) coated specimens, and the corresponding solution pH for (**c**) the substrates and (**d**) coated specimens.

The apatite-forming ability of the coated specimens was evaluated by immersion test in XRD. The concentration of Mg dissolved from the surface, and the pH solution during the immersion test in SBF was monitored. Figure 9 displays the concentration of Mg detected in the solution and the pH solution during 14 days of immersion. The AZ61 alloy released the lowest Mg concentration in the range of 100–150 mg/L indicating the lowest corrosion rate relative to the two Ca-containing alloys, which released 100–500 mg/L Mg into the solution (Figure 9a). The solution pH of the three alloys increased from 7.4 to 8 after one-day immersion indicating high corrosion activities (Figure 9c). The increase in pH occurred due to the release of hydrogen gas during hydrolysis of Mg ions following the corrosion attack. The pH change was not significant as the solution was buffered. The solution pH of AZ61 alloy decreased, approaching the fresh SBF while that of the AZX611 and AZX612 alloys fluctuated with increasing immersion time. On average, the lowest pH was attained in the AZ61 alloy. The PEO-coated specimens released much lower Mg into the solution relative to their substrates (Figure 9b). In agreement, the solution pH of the coated specimen was relatively lower compared to the substrates during the 14-day immersion. The dissolved Mg in the solution was attributed to both corrosion of the underlying substrate and the coating dissolution. The coated AZ61 specimen released 110 to 200 mg/L Mg at the initial immersion time and then stabilized at day 7 to 12. Dissolution no longer occurred at day 14. The coating on AZX611 specimen exhibited a relatively constant dissolution rate during 1–12 days followed by passivation at the end period of the test. The AZX612 specimen exhibited a dynamic dissolution resulting in a highly fluctuating concentration of the dissolved Mg in the range 300–450 mg/L. All of the coatings exhibited the tendency for passivation at day 14 with solution pH at 7.7, which was presumably due to surface coverage by the apatite layers on their surfaces. 

**Figure 10:**
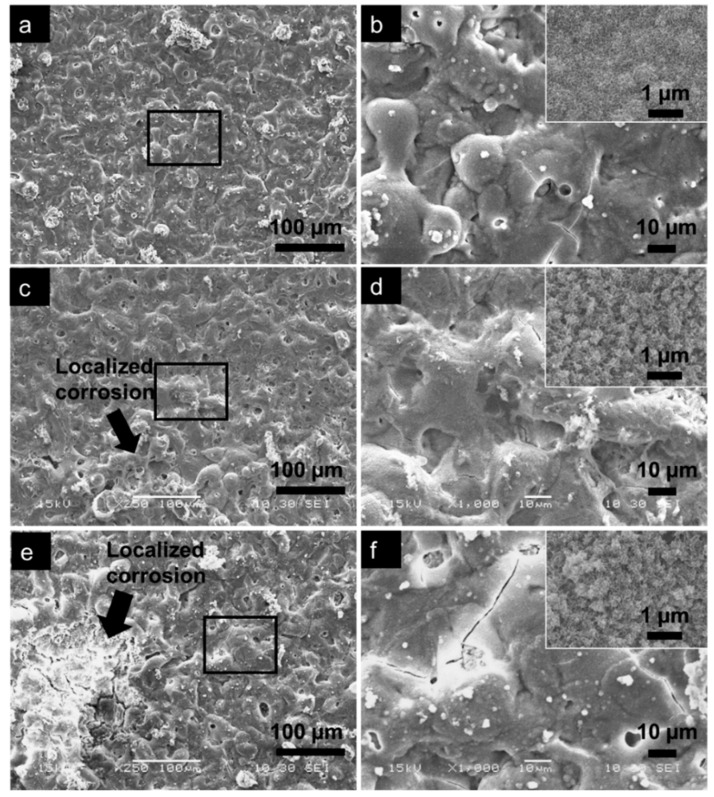
SEM images of PEO coatings on (**a**) and (**b**) AZ61, (**c**) and (**d**) AZX611, and (**e**) and (**f**) AZX612 specimens after 14 days of immersion in SBF showing the corrosion morphology. (b), (d), (f) are higher magnification images of the area inside the rectangle in images (a), (c), (e), respectively. The inset image shows the apatite layer structure.

The presence of apatite on the PEO coating was investigated by observing the surface morphology and analyzing the surface composition after the immersion test. Figure 10 shows the SEM images at different magnifications revealing the presence of uniform apatite layer on all of the three coatings. Most of the pores that existed in the coating of AZ61 specimen were no longer viewed, indicating surface coverage by thick apatite layer (Figure 10a). The layer formed uniformly following the coating structure, as shown clearly in Figure 10b. The spongy structure with scallop type of grain, which was a typical morphology of apatite formed in SBF was seen in a higher magnification image in the inset Figure 10b. The coating remained intact and protected by the apatite layer. Thinner apatite layers were formed on AZX611 and AZX612 specimens as inferred from the existence of open pores and cracks in the coating (Figure 10c,e). The scallop grain of apatite layer in the AZX611 and AZX612 specimens was smaller than that of formed in AZ61 specimen as shown in the inset image of Figure 9d,f. Localized coating damage was recognized at a few spots, pointed by arrows in the images. The AZX611 specimen showed relatively small coating deterioration while larger coating damage on an area approximately 150 µm × 300 µm was observed on AZX612 specimen.

**Figure 11:**
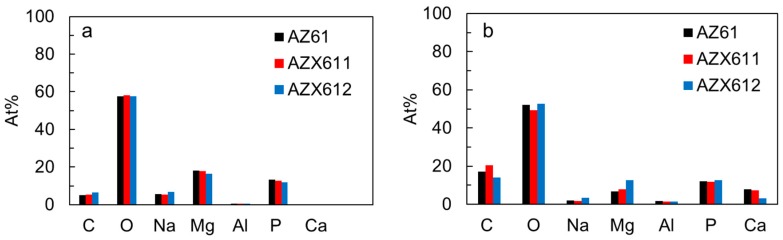
Atomic fraction of elements detected by EDS analysis on PEO coatings of AZ61, AZX611, and AZX612 specimens (**a**) before and (**b**) after 14 days immersion in SBF.

Figure 11 shows the results of EDS analysis from the areas shown in Figure 10 as compared to that of fresh PEO coating. The observation areas for both cases were kept identical by using similar magnification scale. Prior to the immersion test, calcium was not detected in any of the coatings (Figure 11a). The signal attributed to the calcium oxide phase in the coating was weak relative to the other elements. After 14 days immersion in SBF, the Ca signal was detected as high as 8.0, 7.2, and 3.1 at.% in AZ61, AZX611, and AZX612 specimens, respectively, attributed to the apatite layer shown in Figure 10. The Mg concentration increased from 6.8 at.% in the AZ61 to 12.5 at.% in the AZX611 specimens. The concentration of P was relatively not altered at 12–13 at.%. The ratios of Ca/P of the apatite layers were 0.65, 0.60, and 0.24 for the layers formed on AZ61, AZX611, and AZX612 specimens, respectively. The ratio was half of the stoichiometry apatite, 1.67 [30]. The apatite layer that formed in the coating of AZX611 and AZX612 alloys contained more Mg than that of the AZ61 alloy. Moreover, the strong signal of P derived from the underlying PEO coating lowered the Ca/P ratio. 

## 4. Discussion

Surface characterization showed that the coatings formed on all the three alloys exhibited similar uneven structure decorated with pores and cracks, but the composition and thickness were altered by the presence of Ca in the alloys. The coatings composed of a mixed crystalline and amorphous phase. The Mg_3_(PO_4_)_2_ and Mg(PO_3_)_2_ existed in both crystalline and amorphous states as confirmed by XRD analysis, while MgO and Al_2_O_3_ were present in an amorphous state as detected by GD-OES and EDS analysis. The presence of Ca in the alloys induced the formation of amorphous calcium oxide/hydroxide phase in the coatings. The depth profile analysis by GD-OES (Figure 5) confirmed the presence of calcium oxide/hydroxide from the surface throughout the coating-metal interface. At identical anodization time, the resulting coating on the AZX611 alloy was 7 µm thicker than that of the base alloy. The coating did not thicken any further in the AZX612 alloy, as no further substantial change in the Ca content of the solid solution matrix. 

The microstructure observation revealed that the alloying element Ca decreased the density of intermetallic precipitates in the AZ61 alloys by inducing larger precipitates size and enlarging the metallic grain. The grain size expanded about 30–50 times by the presence of 1 wt.% Ca in the alloy. The substitute Ca atom with atomic diameter larger than Mg atom [31] caused expansion in the matrix grain size. The high number of submicron-sized precipitates in the AZ61 alloy transformed into a low number of 10 micron-sized precipitates as a result of alloying with 1 wt.% Ca (Figure 2). The precipitates grew slightly larger, but the metallic grain size remained the same, with increasing Ca concentration to 2 wt.%. The enlargement of grain size was controlled by the amount of dissolved Ca in the solid solution matrix. The precipitates continued to grow as more excess of Ca existed in the AZX612 alloy. The limited solubility of Ca in Mg, which is only 0.8 wt.% [32], induced the formation of Al_2_Ca precipitates. With increasing Ca content, the bigger precipitates grew larger at the expense of the smaller ones following Ostwald ripening [33]. Another intermetallic phase Mg_2_Ca also existed in the AZX611 and AZX612 alloys, but only in a small number. The large misfit in atomic size between Ca and Mg in which the diameter of the Ca atom is 20% larger than Mg, is less favorable for the formation of Mg_2_Ca phase [31]. The presence of heat-resistant Al_2_Ca and Mg_2_Ca phases delayed the formation of strong plasma discharge during electrolytic process resulting in a less crystalline oxide phases in the coatings. Moreover, the presence of Mg_2_Ca phase is not beneficial from the corrosion viewpoints as it is more anodic than the Mg matrix [2].

During the PEO formation, Mg and Ca in the solid solution matrix was selectively oxidized. From thermodynamic viewpoints, Ca is easier to oxidize as it has lower Gibbs free energy than Mg [33]. However, since the concentration of Ca in the solid solution matrix was limited by its solubility, the resulting calcium oxide/hydroxide in the coating was relatively small compared to the magnesium phosphate/oxide phases. The GD-OES depth profile was proved to be a sensitive technique in detecting the calcium oxide/hydroxide. The calcium oxide/hydroxide phases presumably occurred during the 12 min exposure in fine plasma discharge. The strong discharge, which occurred in the latter stage, tended to dissolve the calcium oxide/hydroxide to the solution. The incorporation of calcium oxide/hydroxide contributed to the thickening of the coating formed on AZX611 and AZX612 alloys relative to the AZ61 alloy.

The higher resistivity offered by the thicker coating formed on the AZX611 and AZX612 alloys was counteracted by the vulnerability of their metal substrates to localized corrosion relative to the AZ61 alloy. The polarization coating resistance of the coated AZ61 specimen was higher than that of the AZX611 and AZX612 specimens. The enlargement of precipitates in the AZX611 and AZX612 alloys increased the contact area of galvanic coupling between the precipitates and the matrix, and therefore increased the danger to micro galvanic corrosion when exposed to the corrosive solution. Ennoblement of corrosion potentials in the polarization curves of the AZX611 and AZX612 alloys due to the increase number of cathodic intermetallic was balanced by the increased susceptibility to localized corrosion. The presence of inductive loop in the EIS spectra of AZX611 and AZX612 alloys (Figure 7a), which was not present in the spectra of AZ61 alloy, was attributed to the localized corrosion. A small amount of big precipitates accelerates corrosion, whereas finely distributed precipitates retarded corrosion [34]. The coating–metal interface in the vicinity of the precipitates was less protective. Once the corrosive solution penetrated the coatings, pockets of the solution were developed at the interface. Corrosion of the metal substrate occurred immediately as it was exposed to the solution inside the pockets. The increase in intermetallic precipitates size with Ca content in the alloys contributed to the enlargement of the attacked areas (Figure 10).

The higher corrosion rate of the underlying substrate limited the apatite-forming ability of the coatings on the AZX611 and AZX612 specimens. Even though a uniform apatite layer grew on all of the three coatings as a result of immersion in SBF, the layers that formed on the AZX611 and AZX612 coatings was thinner and contained higher Mg than that formed on the AZ61 coating. The incorporation of calcium oxide/hydroxide in the coatings did not necessarily enhance the bioactivity of the coatings, similar to that reported in Reference [19]. The corrosion rate of the underlying substrate is a more important factor in supporting the growth of apatite. An alternate corrosion rate of the underlying substrate, which released fluctuated hydrogen gas, tends to disturb the deposition of apatite on the coatings. In the case of AM60 alloys [23], the corrosion resistance of the PEO-coated specimens were relatively unaltered, and therefore the formation of apatite was not affected by the variation of Ca content in the alloys. During initial immersion time, thinning of the PEO coating occurred selectively at the amorphous part, leaving a rough crystalline oxide phase. The resulting sub-micron surface roughness became a preferential site for nucleation of apatite [14]. The apatite layer was likely to form on the coatings during 7–14 days immersion time where a constant dissolution rate of Mg was attained (Figure 9b). The released substances from the specimens contributed to increasing the degree of supersaturation of the SBF and the local pH, which triggered the deposition of apatite [30]. 

The following discussion led to the conclusion that the corrosion resistance of the PEO coatings formed on the AZ61 alloys was influenced by the concentration of alloying element Ca, in particular the concentration of the dissolved Ca in the solid solution matrix. Therefore, it is not necessary to add Ca above its solubility in Mg matrix. The Ca-containing precipitates that tended to shorten the lifetime of strong discharge during the PEO process resulted in the formation of lower crystalline oxide phases in the coating. A thinner PEO coating with a high-volume ratio of crystalline to amorphous phases gives much better corrosion resistance than a thicker coating with a lower ratio [12]. Addition of Ca above the solubility limit did not have a beneficial effect on the coating properties. On the contrary, the excess of Ca in the alloys resulted in the enlargement of Al_2_Ca and Mg_2_Ca precipitates, which tended to accelerate localized corrosion. 

## 5. Conclusions

The PEO coating significantly enhanced the corrosion resistance of the Mg-6Al-1Zn-xCa (AZX) alloys relative to their substrates. The presence of 1 and 2 wt.% Ca in the alloys resulted in the formation of thicker coating, which contributed in higher enhancement of the coating resistance. However, the EIS spectra and the surface observation after the immersion test indicated an increased danger to localized corrosion with increasing Ca content in the alloys. The coating formed on AZX alloys exhibited lower polarization resistance than that which formed on the AZ61 alloy. After the 14-day immersion tests in SBF, the coating on AZ61 alloy remained intact while the coatings on AZX611 and AZX612 showed noticeable local damage. The microstructure investigation revealed that the alloying element Ca caused enlargement of metallic grain size and intermetallic precipitates relative to the AZ61 alloy. The increase in corrosion activities underneath the coatings lowered the apatite-forming ability of the AZX coatings, resulting in a thinner apatite layer formed during immersion in SBF.

## Figures and Tables

**Table 1:** Chemical composition of the alloys.

Element	AZ61	AZX611	AZX612
Mg	Bal.	Bal.	Bal.
Al	6.50	5.8–7.2	5.8–7.2
Zn	0.92	0.4–1.5	0.4–1.5
Mn	0.32	0.15–0.5	0.15–0.5
Cu	≤0.05	≤0.05	≤0.05
Ni	≤0.005	≤0.005	≤0.005
Fe	≤0.005	≤0.005	≤0.005
Si	≤0.1	≤0.1	≤0.1
Ca	-	1.0	2.0

**Table 2:** Ionic composition of simulated body fluid (SBF).

Ion	Na^+^	K^+^	Mg^+^	Ca^+^	Cl^−^	HCO_3_^−^	HPO_4_^2−^	SO_4_^2−^
**Concentration (mM)**	142	5	1	2.5	126	10	1	1

**Table 3:** Parameter of the fitted plotting of EIS spectra.

Specimen	Rs (Ωcm^2^)	CPE1 (Ω^−1^s^n^cm^−2^)	n_1_	R1 (Ωcm^2^)	CPE2 (Ω^−1^s^n^cm^−^^2^)	n_2_	R2 (Ωcm^2^)	L (H)	RL (Ωcm^2^)
AZ61	28.25	9.78 × 10^−6^	0.88	1.32 × 10^3^	−1.00 × 10^−3^	0.81	−200	-	-
AZX611	9.40	7.68 × 10^−6^	0.94	1.10 × 10^3^	−1.00 × 10^−3^	0.75	−1000	15	700
AZX612	25.0	8.00 × 10^−6^	0.88	1.00 × 10^3^	−6.00 × 10^−4^	0.75	−725	20	950
AZ61 (Coated)	40.5	1.09 × 10^−9^	0.87	1.04 × 10^4^	3.67 × 10^−7^	0.91	3.61 × 10^3^	-	-
AZX611 (Coated)	61.9	1.64 × 10^−9^	0.87	8.4 × 10^3^	2.48 × 10^−7^	0.96	2.61 × 10^3^	-	-
AZX612 (Coated)	163.8	1.97 × 10^−9^	0.82	9.45 × 10^3^	3.67 × 10^−7^	0.99	1.41 × 10^3^	-	-

## References

[1] Song G. (2007). Control of biodegradation of biocompatable magnesium alloys. Corros. Sci..

[2] Kirkland N., Lespagnol J., Birbilis N., Staiger M., Kirkland N. (2010). A survey of bio-corrosion rates of magnesium alloys. Corros. Sci..

[3] Witte F., Hort N., Vogt C., Cohen S., Ulrich K., Willumeit R., Feyerabend F. (2008). Current Opinion in Solid State and Materials Science Degradable biomaterials based on magnesium corrosion. Curr. Opin. Solid State Mater. Sci..

[4] Patel J.L., Saka N. (2001). Microplasmic ceramic coating. Int. Ceram. Rev..

[5] White L., Koo Y., Neralla S., Sankar J., Yun Y. (2016). Enhanced mechanical properties and increased corrosion resistance of a biodegradable magnesium alloy by plasma electrolytic oxidation (PEO). Mater. Sci. Eng. B.

[6] Echeverry-Rendon M., Duque V., Quintero D., Robledo S.M., Harmsen M.C., Echeverria F. (2018). Improved corrosion resistance of commercially pure magnesium after its modification by plasma electrolytic oxidation with organic additives. J. Biomater. Appl..

[7] Matykina E., Garcia I., Arrabal R., Mohedano M., Mingo B., Sancho J., Merino M., Pardo A. (2016). Role of PEO coatings in long-term biodegradation of a Mg alloy. Appl. Surf. Sci..

[8] Lu J., He X., Li H., Song R. (2018). Microstructure and Corrosion Resistance of PEO Coatings Formed on KBM10 Mg Alloy Pretreated with Nd(NO_3_)_3_. Materials.

[9] Li L., Gao J., Wang Y., Srinivasan P.B., Liang J., Blawert C., Störmer M., Dietzel W., Asoh H., Ono S. (2015). Effect of current density on the microstructure and corrosion behaviour of plasma electrolytic oxidation treated AM50 magnesium alloy. Surf. Coat Technol..

[10] Hussein R., Northwood D., Nie X. (2013). The effect of processing parameters and substrate composition on the corrosion resistance of plasma electrolytic oxidation (PEO) coated magnesium alloys. Surf. Coat. Technol..

[11] Ono S., Moronuki S., Mori Y., Koshi A., Liao J., Asoh H. (2017). Effect of Electrolyte Concentration on the Structure and Corrosion Resistance of Anodic Films Formed on Magnesium through Plasma Electrolytic Oxidation. Electrochim. Acta.

[12] Asoh H., Matsuoka S., Sayama H., Ono S. (2010). Anodizing under sparking of AZ31B magnesium alloy in Na3PO4 solution. J. Jpn. Inst. Light Met..

[13] Kanter B., Vikman A., Brückner T., Schamel M., Gbureck U., Ignatius A. (2018). Bone regeneration capacity of magnesium phosphate cements in a large animal model. Acta Biomater..

[14] Anawati, Asoh H., Ono S. (2015). Enhanced uniformity of apatite coating on a PEO film formed on AZ31 Mg alloy by an alkali pretreatment. Surf. Coatings Technol..

[15] Han Y., Hong S.H., Xu K. (2003). Structure and in vitro bioactivity of titania-based films by micro-arc oxidation. Surf. Coatings Technol..

[16] Gu X., Li N., Zhou W., Zheng Y., Zhao X., Cai Q., Ruan L. (2011). Corrosion resistance and surface biocompatibility of a microarc oxidation coating on a Mg–Ca alloy. Acta Biomater..

[17] Kannan M.B., Mathan B.K. (2016). Electrochemical deposition of calcium phosphates on magnesium and its alloys for improved biodegradation performance: A review. Surf. Coat. Technol..

[18] Gao Y., Yerokhin A., Matthews A. (2015). Deposition and evaluation of duplex hydroxyapatite and plasma electrolytic oxidation coatings on magnesium. Surf. Coat. Technol..

[19] Srinivasan P.B., Liang J., Blawert C., Stormer M., Dietzel W. (2010). Characterization of calcium containing plasma electrolytic oxidation coatings on AM50 magnesium alloy. Appl. Surf. Sci..

[20] Yang J., Lu X., Blawert C., Di S., Zheludkevich M.L. (2017). Microstructure and corrosion behavior of Ca/P coatings prepared on magnesium by plasma electrolytic oxidation. Surf. Coat. Technol..

[21] Lederer S., Sankaran S., Smith T., Fürbeth W. (2019). Formation of bioactive hydroxyapatite-containing titania coatings on CP-Ti 4+ alloy generated by plasma electrolytic oxidation. Surf. Coat. Technol..

[22] Adeleke S., Ramesh S., Bushroa A., Ching Y., Sopyan I., Maleque M., Krishnasamy S., Chandran H., Misran H., Sutharsini U. (2018). The properties of hydroxyapatite ceramic coatings produced by plasma electrolytic oxidation. Ceram. Int..

[23] Anawati A., Asoh H., Ono S. (2017). Effects of alloying element ca on the corrosion behavior and bioactivity of anodic films formed on AM60 mg alloys. Materials.

[24] Anawati A., Asoh H., Ono S. (2018). Degradation Behavior of Coatings Formed by the Plasma Electrolytic Oxidation Technique on AZ61 Magnesium Alloys Containing 0, 1 and 2 wt.% Ca. Int. J. Technol..

[25] Jun J.H., Park B.K., Kim J.M., Kim K.T., Jung W.J. (2009). Effects of Ca Addition on Microstructure and Mechanical Properties of Mg-RE-Zn Casting Alloy. Mater. Sci. Forum.

[26] Jang Y., Tan Z., Jurey C., Xu Z., Dong Z., Collins B., Yun Y., Sankar J. (2015). Understanding corrosion behavior of Mg–Zn–Ca alloys from subcutaneous mouse model: Effect of Zn element concentration and plasma electrolytic oxidation. Mater. Sci. Eng. C.

[27] Brady M.P., Fayek M., Leonard D.N., Meyer H.M., Thomson J.K., Anovitz L.M., Rother G., Song G.L., Davis B. (2017). Tracer Film Growth Study of the Corrosion of Magnesium Alloys AZ31B and ZE10A in 0.01% NaCl Solution. J. Electrochem. Soc..

[28] Channing S. (1991). Annual Book of ASTM Standards.

[29] Müller L., Müller F.A. (2006). Preparation of SBF with different HCO3-content and its influence on the composition of biomimetic apatites. Acta Biomater..

[30] Lu X., Leng Y. (2005). Theoretical analysis of calcium phosphate precipitation in simulated body fluid. Biomaterials.

[31] Pekguleryuz M., Kainer K., Kaya A. (2013). Fundamentals of Magnesium Alloy.

[32] Massalski T., Okamoto H., Subramanian P., Kacprzak L. (1990). Binary Alloy Phase Diagrams.

[33] Hosford W.F. (2010). Physical Metallurgy.

[34] Lunder O., Nordien J., Nisancioglu K. (1997). Corrosion Resistance of Cast Mg-Al Alloys. Corros. Rev..

